# Sodium Selenite Induces Autophagy and Apoptosis in Cervical Cancer Cells via Mitochondrial ROS-Activated AMPK/mTOR/FOXO3a Pathway

**DOI:** 10.3390/antiox13081004

**Published:** 2024-08-19

**Authors:** Cunqi Lv, Qingyu Zeng, Lei Qi, Yuanyuan Wang, Jiacheng Li, Huixin Sun, Linlin Du, Shuxiu Hao, Guijin Li, Chen Feng, Yu Zhang, Cheng Wang, Xinshu Wang, Rong Ma, Tong Wang, Qi Li

**Affiliations:** 1 Institute of Keshan Disease, Chinese Center for Endemic Disease Control, Harbin Medical University, Harbin 150081, China; 2022020106@hrbmu.edu.cn (C.L.);; 2 Key Lab of Etiology and Epidemiology, Education Bureau of Heilongjiang Province, Ministry of Health, Harbin Medical University, Harbin 150081, China; 3 School of Public Health, Qiqihar Medical University, Qiqihar 161003, China; 4 Department of Clinical Medicine, Queen Mary College, Nanchang University, Nanchang 330000, China; 5 Department of Gynecological Oncology, Harbin Medical University Cancer Hospital, Harbin 150081, China; 6 Department of Radiotherapy, Harbin Medical University Cancer Hospital, Harbin 150081, China

**Keywords:** AMPK/mTOR/FOXO3a pathway, cervical cancer, RNA-sequencing, mitochondrial reactive oxygen species, sodium selenite, autophagy

## Abstract

Selenium (Se) is an essential trace element known for its significant role in maintaining human health and mitigating disease progression. Selenium and its compounds exhibit high selective cytotoxicity against tumor cells. However, their anti-cervical cancer (CC) effects and underlying mechanisms have not been fully explored. This study found that sodium selenite (SS) inhibits the viability of HeLa and SiHa cells in a dose- and time-dependent manner. Intraperitoneal injection of 3 and 6 mg/kg SS for 14 days in female nude mice significantly inhibited the growth of HeLa cell xenografts without evident hepatotoxicity or nephrotoxicity. RNA sequencing results indicated that the AMP-activated protein kinase (AMPK), Forkhead box protein O (FOXO), and apoptosis signaling pathways are key regulatory pathways in SS’s anti-CC effects, and SS’s inhibition of HeLa cell proliferation may be related to autophagy and ROS-induced apoptosis. Further research has revealed that SS induces cell autophagy and apoptosis through the AMPK/mTOR/FOXO3a pathway, characterized by the upregulation of p-AMPK/AMPK, FOXO3a, LC3-II, cleaved-caspase3, and cleaved-PARP and the downregulation of p-mTOR/mTOR and p62. Additionally, SS impaired mitochondrial function, including decreased mitochondrial membrane potential, mitochondrial Ca^2+^ overload, and accumulation of mitochondrial reactive oxygen species (mtROS). Pretreatment with Mitoquinone mesylate (Mito Q) and compound C partially reversed SS-induced apoptosis, autophagy, and proliferation inhibition. Pretreatment with 3-methyladenine (3-MA) enhances SS-induced apoptosis and proliferation inhibition in HeLa cells but reverses these effects in SiHa cells. In summary, SS induces apoptosis, autophagy, and proliferation inhibition in HeLa and SiHa cells through the activation of the AMPK/mTOR/FOXO3a signaling pathway via mtROS. Autophagy activation may be a major risk factor for SS-induced apoptosis in SiHa cells but can protect HeLa cells from SS-induced apoptosis. These findings provide new evidence for understanding the molecular mechanisms underlying SS in potential new drug development for CC.

## 1. Introduction

Cervical cancer (CC) is one of the most common malignancies of the female reproductive system [1]. CC accounts for approximately 600,000 new cases and 342,000 deaths worldwide each year [2], with the majority occurring in resource-limited areas [3]. As one of the primary treatments for CC, radiotherapy can significantly improve the prognosis of CC. However, tumor radioresistance greatly limits the overall efficacy of radiotherapy in treating CC [4]. Therefore, there is an urgent need to develop novel therapeutic drugs for the treatment of CC.

Selenium (Se) is an essential trace element for human health with important biological functions [5]. Epidemiological studies have shown that reduced serum selenium levels are significantly associated with the incidence of endometrial cancer and breast cancer [6,7,8]. Sodium selenite (SS), the most extensively studied inorganic selenium compound, has been demonstrated to exhibit anti-cancer activity in various cancer cell lines [9,10,11,12,13,14,15]. Cancer cells are more susceptible to reactive oxygen species (ROS) due to their high metabolic activity and redox imbalance [16]. At supranutritional doses, SS acts as a pro-oxidant, inducing excessive ROS production in tumor cells, thereby altering their redox homeostasis and exerting cytotoxic effects [17]. The induction of oxidative stress plays a critical role in the anti-tumor effects of SS.

Autophagy and apoptosis are two key pathways in cell survival and death, playing crucial roles in tumor development and progression. Under many stress conditions, both autophagy and apoptosis are induced, controlling cell survival or death. Generally, autophagy can block the induction of apoptosis, counteracting ROS-mediated damage in a cell-protective manner [18]. However, in some cases, autophagy may induce apoptosis or necrosis, leading to autophagic cell death through excessive degradation of the cytoplasm [19,20]. SS has been shown to induce autophagy and apoptosis in cancer cells via PI3K/Akt/mTOR, but most of these studies have focused on areas such as colorectal and gastric cancer [21,22]. No reports are available on supranutritional doses of selenium inducing CC autophagy. Therefore, more comprehensive studies are needed to elucidate the role of SS in CC and its possible mechanisms.

Based on the above background, the aim of this study was to investigate the mechanism of SS action in HeLa and SiHa cells, including oxidative stress, autophagy, and apoptosis, by in vivo and in vitro assays. Meanwhile, the relationship between autophagy and apoptosis in SS toxicology is not clear. The role of autophagy in SS-induced apoptosis (promotion or inhibition) in HeLa and SiHa cells also remains to be investigated.

## 2. Materials and Methods

### 2.1. Cell Culture and Chemicals

Human cervical cancer (CC) cell lines, HeLa and SiHa, were obtained from the Cell Bank of the Chinese Academy of Sciences (Shanghai, China). Cells were cultured in RPMI-1640 and DMEM medium (Gibco, Grand Island, NY, USA) containing 10% fetal bovine serum (FBS), 10 µg/mL streptomycin, and 100 U/mL penicillin, incubated at 37 °C with 5% CO_2_. Sodium selenite (SS) and Chloroquine diphosphate salt (CQ) were purchased from Sigma-Aldrich (St. Louis, MO, USA). Furthermore, 3-methyladenine (3-MA), Mitoquinone mesylate (Mito Q), and Rhod-2 AM were obtained from MCE (Monmouth Junction, NJ, USA). Compound C, Cell Counting Kit-8 (CCK-8), Annexin V-FITC/PI Apoptosis Kit, and 2-NBDG were provided by ApexBio (Houston, TX, USA). MitoSOX Red and Trizol were purchased from Invitrogen (Carlsbad, CA, USA). The JC-1 mitochondrial membrane potential assay kit was provided by Solarbio (Beijing, China). Ad-mCherry-GFP-LC3B, Autophagy Staining Assay Kit with MDC (MDC), and ATP Assay Kit were obtained from Beyotime (Shanghai, China). The ADP Fluorescence Detection Kit was purchased from Elascience (Wuhan, China). 5(6)-Carboxyfluorescein diacetate succinimidyl ester (5(6)-CFDA SE; CFSE) was obtained from Absin (Shanghai, China).

Primary antibodies against p-AMPK (#2535), AMPK (#5831), p-mTOR (#5536), mTOR (#2983), p62 (#8025), and cleaved-caspase3 (#9661), and HRP-conjugated secondary antibodies (#4412) were obtained from Cell Signaling Technology (Danvers, MA, USA). Primary antibodies against FOXO3a (#ab47285), LC3B (#ab192890), and cleaved-PARP (#ab32064) were purchased from Abcam (Cambridge, UK).

### 2.2. Cell Viability Assay

Cell viability was assessed using the CCK-8 method. Cells were seeded at a density of 5000 cells/well in a 96-well plate and incubated for 12 h. The cells were then treated with varying concentrations of SS at 0, 2.5, 5, 7.5, 10, 15, 20, and 40 µM for 6, 12, and 24 h. After treatment, CCK-8 solution was mixed with the culture medium at a 1:10 volume ratio and incubated at 37 °C for 60 min. Absorbance was measured at 450 nm using a microplate reader (BioTek Cytation 3, BioTek, Winooski, VT, USA). The half maximal inhibitory concentration (IC50) values for SS in each cell line were calculated using GraphPad Prism 8.0.2 software.

### 2.3. Cell Apoptosis

Apoptosis was analyzed using the Annexin V-FITC/PI Apoptosis Kit. Cells were seeded evenly in a six-well plate and subjected to the appropriate treatments. After 24 h of intervention, cells were collected and washed twice with PBS. Cells were resuspended in 500 µL of Binding Buffer and stained with 5 µL of Annexin V-FITC and 5 µL of PI. After mixing, the cells were incubated in the dark for 15 min. Data were collected using a flow cytometer (AccuriTM C6 Plus, BD Biosciences, San Jose, CA, USA) and analyzed with Flow Jo V10.0.7 software.

### 2.4. Cell Proliferation

Cell proliferation was assessed using CFSE staining. Cells were uniformly seeded into six-well plates and treated according to experimental requirements. After 24 h of appropriate intervention, cells were resuspended in 0.5 µM CFSE staining solution and incubated in the dark at 37 °C for 30 min. After staining, cells were washed twice with PBS and divided into three groups: control (no intervention, incubated in complete medium for 48 h post-staining), positive control (no intervention, directly analyzed post-washing), and treatment groups (treated cells, incubated in complete medium for 48 h post-staining). The positive control cells were analyzed immediately after washing, while the control and treatment groups were resuspended in complete medium containing 10% FBS and incubated for 48 h before cell collection. Data were collected using a flow cytometer (FACS Melody, BD Biosciences, San Jose, CA, USA) and analyzed using GraphPad Prism 8.0.2 software.

### 2.5. Cell Migration and Invasion Assays

A Transwell insert with 8 μm pores (Corning) was used to access the migratory and invasive abilities of HeLa cells. A total of 1 × 10^4^ cells was cultured in the upper chamber with 200 μL of serum-free 1640, and 600 μL of medium containing 20% FBS was added to the lower chamber. Following 24 h incubation, the migrated cells were fixed with 4% paraformaldehyde and stained by 0.5% crystal violet for 30 min. For the invasion assay, Transwell chambers were pretreated with 80 μL of Matrigel (BD Biosciences, San Jose, CA, USA), and the remaining steps were the same as with the migration assay. Ultimately, cells were visualized and photographed under a microscope (Olympus, Tokyo, Japan), and the number of cells in each region was calculated by Image J 1.53t software.

### 2.6. Western Blot Analysis

Cells or tissues were lysed using pre-cooled RIPA buffer. Lysates were centrifuged at 12,000× *g* and 4 °C for 15 min, and proteins were separated by SDS-PAGE and transferred onto PVDF membranes. Membranes were blocked with 5% BSA at room temperature for 1 h, followed by overnight incubation with primary antibodies at 4 °C. After three washes with TBST, membranes were incubated with HRP-conjugated secondary antibodies at room temperature for 1 h. Protein bands were visualized using an enhanced chemiluminescence substrate, and images were captured using a Tanon-5200 system (Tanon, Shanghai, China). Results were expressed as relative light density and analyzed using Image J 1.53t software.

### 2.7. Animal Experiments

All animal experiments were conducted in accordance with the Guide for the Care and Use of Laboratory Animals, approved by the Institutional Animal Care and Use Committee of Harbin Medical University (approval number: hrbmuecdc20220401). Five-week-old female BALB/c nude mice were purchased from Vital River Laboratory Animal Technology Co., Ltd. (Beijing, China) and housed in a pathogen-free environment. After a 7-day acclimatization period, HeLa cells in the logarithmic growth phase (1 × 10^6^ cells) were resuspended in 100 µL of PBS and subcutaneously injected into the flanks of the mice. Tumor volume was measured every two days and calculated using the formula: V (mm^3^) = (L × W^2^)/2, where L and W are the length and width of the tumor, respectively. When tumor volumes reached 40–100 mm^3^, mice were randomly assigned to three groups (*n* = 5/group): vehicle control (saline), low-dose SS (3 mg/kg), and high-dose SS (6 mg/kg), administered intraperitoneally every other day. Fourteen days later, mice were euthanized under isoflurane anesthesia, and tumors, liver, and kidney tissues were collected for analysis and fixed in 4% formaldehyde or stored in liquid nitrogen.

### 2.8. Immunohistochemistry and Histopathology

Excised tumor tissues were fixed in 4% paraformaldehyde for 2 days, embedded in paraffin, and sectioned at 4 µm. Sections were deparaffinized and rehydrated, followed by antigen retrieval. Sections were incubated with anti-FOXO3a (1:200) antibody overnight at 4 °C, then with secondary antibodies at 37 °C for 1 h. The reaction was visualized using diaminobenzidine, counterstained with hematoxylin, and examined under an inverted phase-contrast microscope. Quantification was performed using ImageJ, and statistical analysis was conducted with GraphPad Prism 8.0.2 software. Toxicity effects of SS on liver, kidney, and xenograft tumors were evaluated by Hematoxylin and Eosin (H&E) staining.

### 2.9. Immunofluorescence Staining

The prepared dehydrated sections were incubated overnight at 4 °C with anti-LC3B (1:200) and cleaved-caspase3 (1:100) antibodies, followed by incubation with secondary antibodies at 37 °C for 1 h. Samples were counterstained with DAPI and observed using a confocal microscope (LSM 800, Zeiss, Oberkochen, Germany). For cell immunofluorescence staining, cells were fixed in 4% paraformaldehyde, permeabilized, and stained with anti-FOXO3a (1:200) antibody for 15 h. After washing with PBS, cells were incubated with secondary antibodies for 1 h, and images were captured using a confocal microscope (LSM 800, Zeiss, Germany).

### 2.10. RNA Sequencing (RNA-Seq)

RNA-seq analysis was performed by LC-Bio Technologies Co., Ltd. (Hangzhou, China). Total RNA was extracted from cells (*n* = 3/group) using Trizol. High-quality RNA samples were converted into cDNA libraries, and products were enriched and purified with 12–15 cycles of PCR to create the cDNA library. Sequencing was conducted on the Illumina Hiseq 4000 platform. Differentially expressed genes (DEGs) were identified with a fold change > 1.5 and *p* < 0.05.

### 2.11. Measurement of Mitochondrial Superoxide and Membrane Potential (MMP)

Mitochondrial ROS (mtROS) levels were measured using MitoSOX Red. Cells were washed twice with PBS and incubated with 5 µM of MitoSOX Red for 30 min. After washing with PBS, mtROS were detected by confocal microscopy (LSM 800, Zeiss, Germany) and flow cytometry (Accuri^TM^ C6 Plus, BD Biosciences, USA). Comparison was performed based on red fluorescence intensity or changes in fluorescence peak. MMP was assessed using the JC-1 MMP Assay Kit, according to the manufacturer’s instructions, and analyzed by confocal microscopy (LSM 800, Zeiss, Germany) and flow cytometry (Accuri^TM^ C6 Plus, BD Biosciences, USA). Quantification was performed by measuring the fluorescence intensity ratio of aggregate to monomer (red/green). Flow cytometry results were quantified using Flow Jo V10.0.7 and statistically analyzed with GraphPad Prism 8.0.2 software.

### 2.12. Ad-mCherry-GFP-LC3B Infection Analysis

Autophagic flux changes were assessed using Ad-mCherry-GFP-LC3B. Cells were seeded in confocal dishes and incubated for 24 h to achieve 40–50% confluence. Cells were then transfected with Ad-mCherry-GFP-LC3B at 20 MOI for 24 h in medium containing 10% FBS, followed by fresh medium. After various interventions, changes in green and red fluorescence were observed using a confocal microscope (LSM 800, Zeiss, Germany). In non-autophagic conditions, mCherry-GFP-LC3B exhibits diffuse yellow fluorescence (a combination of mCherry and GFP) in the cytoplasm under confocal microscopy. During autophagy, mCherry-GFP-LC3B aggregates on the autophagosome membrane, appearing as yellow puncta under fluorescence microscopy. When autophagosomes fuse with lysosomes, the partial quenching of GFP fluorescence results in the appearance of red puncta.

### 2.13. Flow Cytometric Analysis of Autophagy

Autophagy was measured using MDC staining. MDC is an acidophilic fluorescent probe that specifically binds to autophagosomes and autolysosomes. Cells were seeded in six-well plates and resuspended in MDC working solution after treatment. After 30 min of incubation at 37 °C in the dark, cells were analyzed by flow cytometry (FACS Melody, BD Biosciences, San Jose, CA, USA) and statistically analyzed with GraphPad Prism 8.0.2 software.

### 2.14. Glucose Uptake Assay

Glucose uptake was measured using the fluorescent glucose analog 2-NBDG. Moreover, 2-NBDG is a green fluorescent glucose analog. Since 2-NBDG competes with glucose for the same transport pathways, the uptake of 2-NBDG by cells reflects their glucose uptake activity. By observing the green fluorescence within the stained cells, the glucose uptake capacity of the cells can be assessed. Cells were seeded in confocal dishes and treated accordingly. Each well was incubated with 15 µM of 2-NBDG for 15 min at 37 °C in the dark. After washing with PBS, glucose uptake was detected using a confocal microscope (LSM 800, Zeiss, Germany).

### 2.15. Measurement of Intracellular Ca^2+^

Intracellular Ca^2+^ levels were measured using Rhod-2 AM. Rhod-2 can remain inside the cell and bind to calcium ions, emitting red fluorescence. By measuring the intensity of the red fluorescence within the cells, the intracellular Ca^2^⁺ concentration can be assessed. Cells were seeded in six-well plates or confocal dishes and treated with different concentrations of SS for 24 h. After washing with PBS, cells were incubated with 5 µM of Rhod-2 AM for 30 min at 37 °C in the dark. Ca^2+^ levels were visualized using a confocal microscope (LSM 800, Zeiss, Germany) and measured by flow cytometry (FACS Melody, BD Biosciences, USA) and statistically analyzed with GraphPad Prism 8.0.2 software.

### 2.16. Measurement of ATP and ADP

The ratio of ATP/ADP concentrations was measured by the ATP Assay Kit and the ADP Fluorescence Detection Kit according to the manufacturer’s protocols.

### 2.17. Statistical Analysis

Data are expressed as mean ± standard deviation (SD). The statistical analysis was performed on the data using one-way ANOVA in GraphPad Prism 8.0.2 (GraphPad Software, San Diego, CA, USA) software. The *p*-values less than 0.05 were considered statistically significant.

## 3. Results

### 3.1. SS Inhibits the Proliferation, Migration, and Invasion of Cervical Cancer Cells

**Figure 1:**
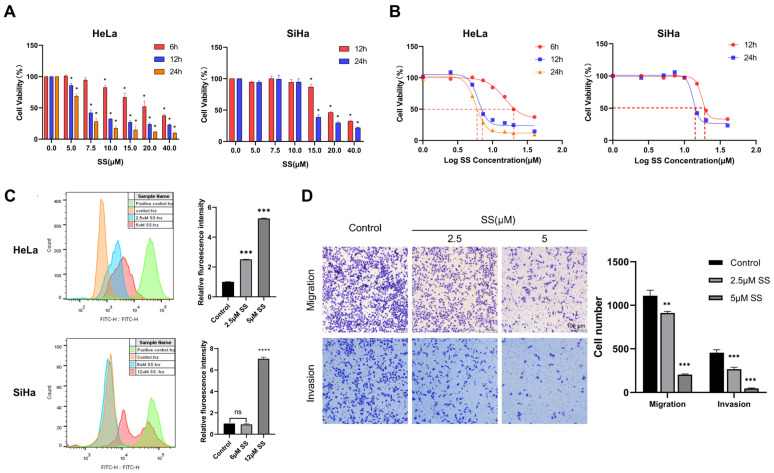
The inhibitory effects of sodium selenite on HeLa and SiHa cell proliferation, migration, and invasion. (**A**,**B**) Cell viability was assessed using the CCK-8 assay after treating cells with various concentrations of SS (0, 2.5, 5, 7.5, 10, 15, 20, and 40 µM) for 6, 12, and 24 h. (**C**) The proliferation ability of HeLa and SiHa cells was assessed using CFSE staining. (**D**) Migration and invasion abilities were evaluated using transwell assays in HeLa cells and quantitatively analyzed by image J. Scale bar, 100 µm. All experiments were repeated at least three times. * indicates statistical significance compared to the control group, * *p* < 0.05, ** *p* < 0.01, *** *p* < 0.001, **** *p* < 0.0001. SS: sodium selenite.

CCK-8 analysis was used to assess the viability of HeLa and SiHa cells after intervention with different concentrations of SS for 6, 12, and 24 h. The results showed that SS significantly inhibited the growth of HeLa and SiHa cells in a time- and dose-dependent manner; the IC50 values of the SS intervention in HeLa cells at 6, 12, and 24 h were 14.81 µM, 6.23 µM, and 5.70 µM, respectively. For SiHa cells, the IC50 values of the SS intervention at 12 and 24 h were 17.50 µM and 13.23 µM, respectively (Figure 1A,B). Therefore, we selected a concentration of 5 µM for HeLa cells and 12 µM for SiHa cells to treat for 24 h in subsequent experiments. HeLa cells are well-known for their high malignancy, rapid proliferation, and significant migration and invasion capabilities. To investigate the effect of SS on the migration and invasion abilities of HeLa cells, we conducted Transwell assays. The results showed that with increasing concentrations of SS, the migration and invasion capabilities of HeLa cells were significantly inhibited (Figure 1D). Furthermore, we employed the CFSE method to assess the impact of SS on the proliferation of HeLa and SiHa cells. As shown in Figure 1C, SS significantly inhibited the reduction in mean CFSE fluorescence intensity in HeLa and SiHa cells, indicating a significant inhibition of their proliferative abilities.

### 3.2. SS Inhibits the Growth of HeLa Cell Xenograft Tumors in Nude Mice

**Figure 2:**
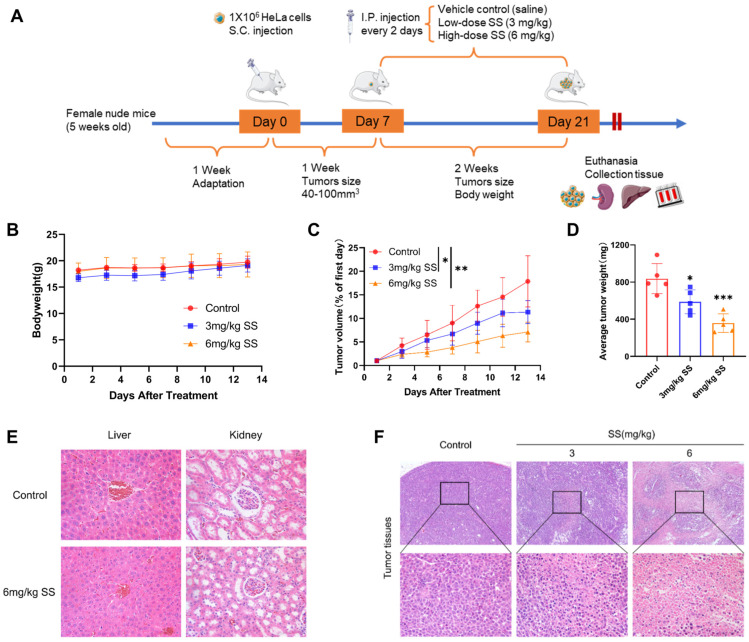
Inhibitory effect of sodium selenite on the growth of HeLa xenografts. (**A**) Schematic diagram of the establishment of HeLa xenograft models and administration (n = 5/group). (**B**) Body weights of the mice were monitored during the experiments for toxicity. (**C**) Tumor volumes were measured by vernier calipers and calculated by the length and width every 2 days. (**D**) Tumor weight in different intervention groups (**E**) Representative histological images of liver and kidney H&E staining in control and 6 mg/kg SS-treated groups, magnification ×400. (**F**) H&E staining of xenografts from control, 3 mg/kg SS, and 6 mg/kg SS intervention groups, magnification ×400. All experimental results were repeated at least five times. * indicates compared to the control group, * *p* < 0.05, ** *p* < 0.01, *** *p* < 0.001. SS: sodium selenite.

To investigate the effect of SS on tumor formation in CC in vivo, a xenograft tumor model was established by injecting HeLa cells into nude mice (Figure 2A). Mice in all intervention groups maintained normal weight gain throughout the intervention period (Figure 2B). Compared to the vehicle control group, tumor volumes in the 3 mg/kg and 6 mg/kg SS intervention groups were inhibited by 36.5% and 60.2%, respectively (Figure 2C), and the tumor weights were inhibited by 29.7% and 57.1%, respectively (Figure 2D). Long-term exposure to or ingestion of excessive amounts of SS may lead to liver dysfunction and kidney damage. To assess whether the current dosage of SS causes liver and kidney damage in nude mice, we employed H&E staining techniques. H&E staining results showed no significant liver or kidney damage in the 6 mg/kg SS intervention group compared to the control group (Figure 2E). Additionally, a large amount of nuclear fragmentation and exfoliation was observed in the tumor tissues of the 3 mg/kg and 6 mg/kg SS intervention groups (Figure 2F).

### 3.3. SS Induces Autophagy Activation and Promotes Apoptosis

**Figure 3:**
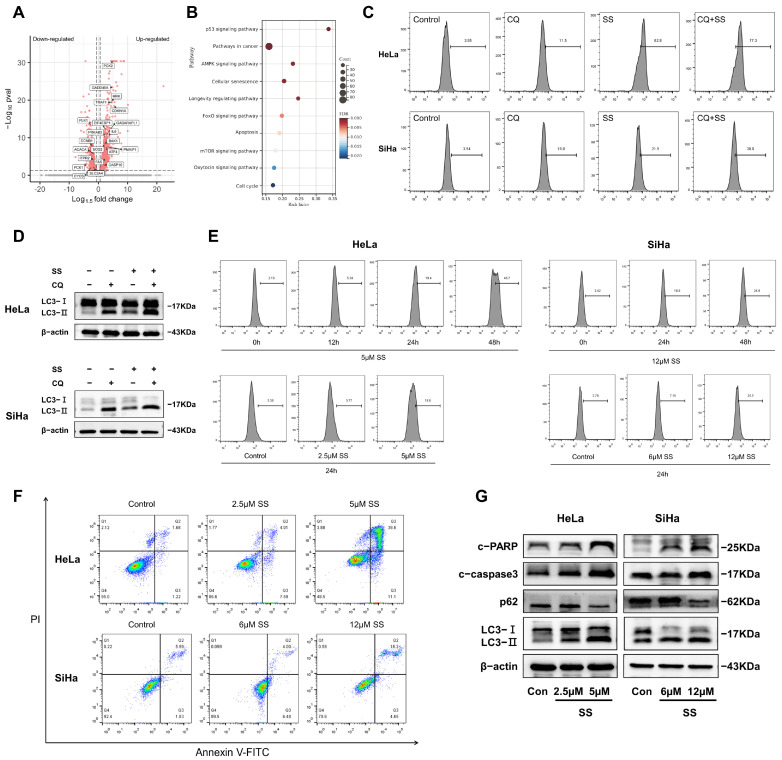
Sodium selenite induces autophagy and apoptosis in HeLa and SiHa cells. (**A**) Volcano plot displaying the distribution of DEGs, highlighting significant genes in AMPK, FOXO, and apoptosis signaling pathways. (**B**) KEGG enrichment analysis showing significant enrichment of DEGs in AMPK, FOXO, and apoptosis pathways. (**C**) Quantitative analysis of the change in the percentage of MDC-positive cells after CQ pretreatment was performed using flow cytometry. (**D**) Changes in LC3-II protein levels after CQ pretreatment. (**E**) Quantitative analysis of the percentage of MDC-positive cells after SS treatment at different times and doses. (**F**) The percentage of apoptotic cells was detected and quantified using flow cytometry. (**G**) Protein expression levels of autophagy markers (LC3-II and p62) and apoptosis markers (cleaved-caspase3 and cleaved-PARP). All experiments were repeated at least three times. Con: Control; SS: sodium selenite; CQ: Chloroquine diphosphate salt; c-PARP: cleaved-PARP; c-caspase3: cleaved-caspase3.

To further explore the mechanism by which SS inhibits HeLa and SiHa cell progression, we performed high-throughput RNA sequencing (RNA-Seq) on SS-treated HeLa cells. The results revealed that 1489 genes were significantly upregulated, while 839 genes were significantly downregulated in SS-treated HeLa cells (Figure 3A). Kyoto Encyclopedia of Genes and Genomes (KEGG) enrichment analysis indicated significant upregulation of AMPK, FOXO, and apoptosis signaling pathways (Figure 3B). Gene Ontology (GO) enrichment analysis showed that these differential genes were assigned to 516, 80, and 131 GO terms involved in biological processes, molecular functions, and cellular components, respectively, many of which are associated with apoptosis, autophagy, ROS production, and Ca^2+^ homeostasis (Figure S1). These results suggested that SS has potential regulatory roles in modulating autophagy, Ca^2+^ signaling pathways, oxidative stress, and inducing apoptosis. Considering these factors, we hypothesize that SS may inhibit HeLa and SiHa cell proliferation by inducing autophagy and apoptosis through the AMPK/mTOR/FOXO3a signaling pathway.

To verify whether SS can induce functional autophagy in HeLa and SiHa cells, we inhibited lysosomal enzyme activity by adding CQ. Western blot results showed that LC3-II expression significantly increased in the CQ-treated group compared to the control group. Moreover, the CQ+SS co-treatment group further promoted LC3-II expression compared to the CQ-treated group (Figure 3D). Next, we assessed autophagic flux using Ad-mCherry-GFP-LC3B in HeLa cells. The results indicated that GFP fluorescence significantly increased in the CQ-treated group compared to the control group, while it was significantly quenched in the SS-treated group. Notably, the CQ+SS co-treatment group not only reversed the SS-induced GFP fluorescence quenching but also further enhanced GFP fluorescence compared to the CQ-treated group (Figure S2B). The MDC staining results further corroborated these findings (Figure 3C and Figure S2C). These results indicate that SS can significantly induce functional autophagy in HeLa and SiHa cells. To further evaluate the impact of SS on autophagy, we examined the effect of different doses and intervention times of SS on the percentage of MDC-positive cells in HeLa and SiHa cells. The percentages of MDC-positive cells after treatment of HeLa cells with 5 µM SS for 0, 12, 24, and 48 h were 2.16%, 5.34%, 19.4%, and 46.7%, respectively. After treatment of HeLa cells with 0, 2.5, and 5 µM SS for 24 h, the percentages of MDC-positive cells were 3.39%, 3.77%, and 19.6%, respectively (Figure 3E and Figure S2D), and similar dose- and time-dependent increases in the percentage of MDC-positive cells were observed in SiHa cells. Additionally, we measured the impact of SS on HeLa and SiHa cell apoptosis levels. As shown in Figure 3F and Figure S2E, compared to the control group, the percentage of apoptotic HeLa and SiHa cells significantly increased with the increasing doses of SS treatment. The expression of LC3-II, p62, cleaved-caspase3, and cleaved-PARP proteins also in HeLa and SiHa cells supported these findings (Figure 3G and Figure S2F).

### 3.4. SS Induces Proliferation Inhibition, Autophagy, and Apoptosis through the AMPK/mTOR/FOXO3a Signaling Pathway

**Figure 4:**
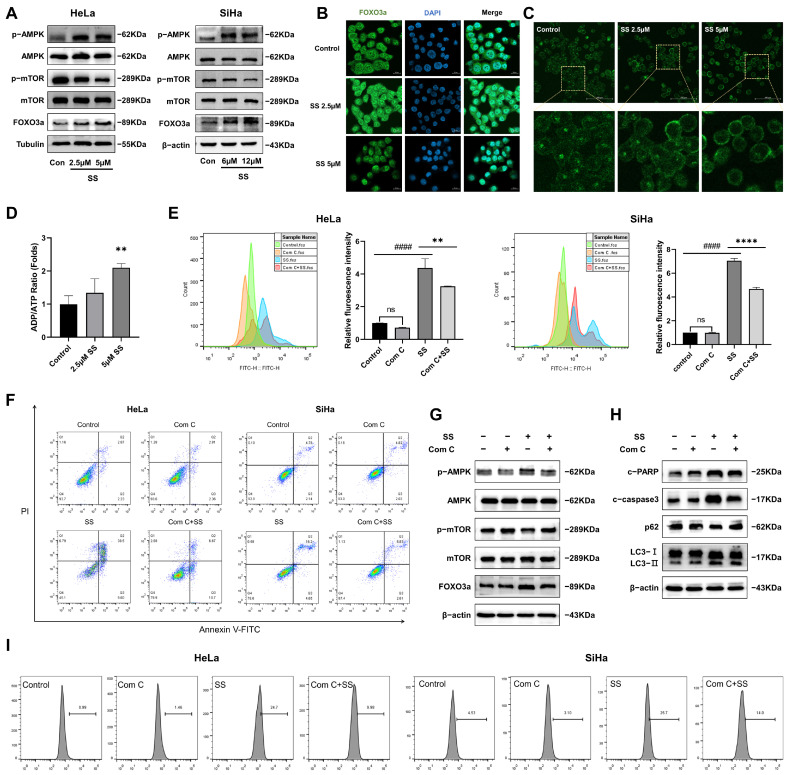
Sodium selenite induces proliferation inhibition, autophagy, and apoptosis via the AMPK/mTOR/FOXO3a pathway in HeLa and SiHa cells. (**A**) Protein expression levels in the AMPK/mTOR/FOXO3a pathway in HeLa and SiHa cells. (**B**) Localization of FOXO3a in the nucleus observed by confocal microscopy in HeLa cells. (**C**) Evaluation of glucose uptake ability using the 2-NBDG fluorescent glucose analog by confocal microscopy in HeLa cells. (**D**) Changes in the ADP/ATP ratio in HeLa cells. Changes in cell proliferation (**E**), the percentage of apoptotic cells (**F**), and the percentage of MDC-positive cells (**I**) in HeLa and SiHa cells after compound C pretreatment. (**G**) Expression of AMPK/mTOR/FOXO3a pathway proteins after compound C pretreatment in HeLa cells. (**H**) Analysis of autophagy- and apoptosis-related protein expression levels in HeLa cells. All results were repeated at least three times. # indicates statistical significance compared to the control group, ns: *p* > 0.05, #### *p* < 0.0001; * indicates statistical significance compared to the SS treatment group, ** *p* < 0.01, **** *p* < 0.0001. Con: Control; SS: sodium selenite; Com C: Compound C, c-PARP: cleaved-PARP; c-caspase3: cleaved-caspase3.

The AMPK/mTOR signaling pathway is recognized as an upstream regulatory pathway of autophagy [23]. As a nuclear transcription factor, FOXO3a can induce autophagy and apoptosis after nuclear translocation [24]. Therefore, the AMPK/mTOR/FOXO3a signaling pathway is likely a key regulatory pathway through which SS induces proliferation inhibition, autophagy, and apoptosis. Our study shows that with increasing doses of SS, the expression levels of p-AMPK and FOXO3a significantly increased, while p-mTOR expression levels significantly decreased in HeLa and SiHa cells (Figure 4A and Figure S3A). To determine whether SS intervention induces nuclear translocation of FOXO3a, we examined the localization of FOXO3a expression using immunofluorescence. The results showed that, compared to the control group, FOXO3a showed significant nuclear translocation in the 5 µM SS treatment group in HeLa cells (Figure 4B). Additionally, AMPK is activated when cellular energy levels decline, a situation particularly evident during glucose starvation [25]. Therefore, we used 2-NBDG to further investigate the effect of SS on glucose uptake. We found that in the control group, the 2-NBDG fluorescence signal was predominantly expressed within the cells, indicating strong glucose uptake capability. In contrast, the intracellular fluorescence signal was reduced in the SS intervention group. This suggests that SS intervention significantly inhibits the glucose uptake capability of HeLa cells (Figure 4C). Furthermore, SS inhibits the uptake of external glucose, raising the question of whether intracellular energy balance is disrupted. Therefore, we investigated the changes in the ADP/ATP ratio. Compared to the control group, the ADP/ATP ratio in HeLa cells treated with SS was significantly increased (Figure 4D).

To confirm the role of AMPK activation in SS-induced proliferation inhibition, autophagy, and apoptosis in HeLa and SiHa cells, we pretreated the cells with compound C. The results showed that co-treatment with compound C and SS effectively reversed AMPK phosphorylation and partially reversed the expression of p-mTOR and FOXO3a in HeLa cells (Figure 4G and Figure S3B). Additionally, compound C reversed SS-induced proliferation inhibition (Figure 4E) and apoptosis levels (Figure 4F and Figure S3C) in HeLa and SiHa cells. Compared to the SS treatment group, pretreatment with compound C significantly reduced the percentage of MDC-positive cells (Figure 4I and Figure S3E) and quenched the green fluorescence of Ad-mCherry-GFP-LC3B (Figure S3F). The expression levels of LC3-II, p62, cleaved-caspase3, and cleaved-PARP were also significantly reversed by compound C in HeLa cells (Figure 4H and Figure S3D). These results suggest that SS inhibits cell proliferation and induces autophagy and apoptosis by suppressing glucose uptake and reducing the ADP/ATP ratio, thereby activating the AMPK/mTOR/FOXO3a pathway.

### 3.5. SS Inhibits Proliferation and Induces Autophagy and Apoptosis in Cervical Cancer Cells via mtROS

**Figure 5:**
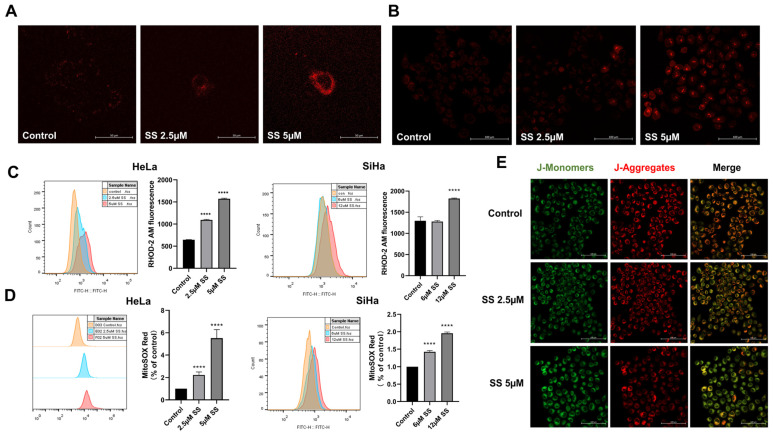
Effects of sodium selenite on Ca^2+^ levels, mitochondrial ROS production, and membrane potential in HeLa and SiHa cells. (**A**,**B**) Observation of intracellular Ca^2+^ (**A**, magnification ×400) and mtROS (**B**, magnification ×200) levels by confocal microscopy in HeLa cells. (**C**,**D**) Quantitative analysis of Ca^2+^ (**C**) and mtROS (**D**) levels in HeLa and SiHa cells using flow cytometry. (**E**) The levels of MMP were evaluated using JC-1 staining, visualized with a confocal microscope, magnification ×200. All results were repeated at least three times. * indicates statistical significance compared to the control group, **** *p* < 0.0001.

GO and KEGG enrichment analyses showed that SS intervention is associated with ROS production and Ca^2+^ homeostasis. Mitochondria can uptake and store intracellular Ca^2+^, particularly Ca^2+^ originating from the endoplasmic reticulum. Excessive Ca^2+^ can lead to a decrease in mitochondrial membrane potential, increased production of peroxides, and mitochondrial damage, which may result in cellular dysfunction or apoptosis [26]. Therefore, we examined mitochondrial Ca^2+^ and mtROS levels. The results demonstrated that SS significantly induced mitochondrial Ca^2+^ overload (Figure 5A,C) and mtROS accumulation (Figure 5B,D). We further investigated the effect of SS on mitochondrial function using JC-1 staining in HeLa cells. Compared to the control group, increasing intervention doses resulted in a significant decrease in red fluorescence intensity and an increase in green fluorescence intensity (Figure 5E and Figure S4A).

**Figure 6:**
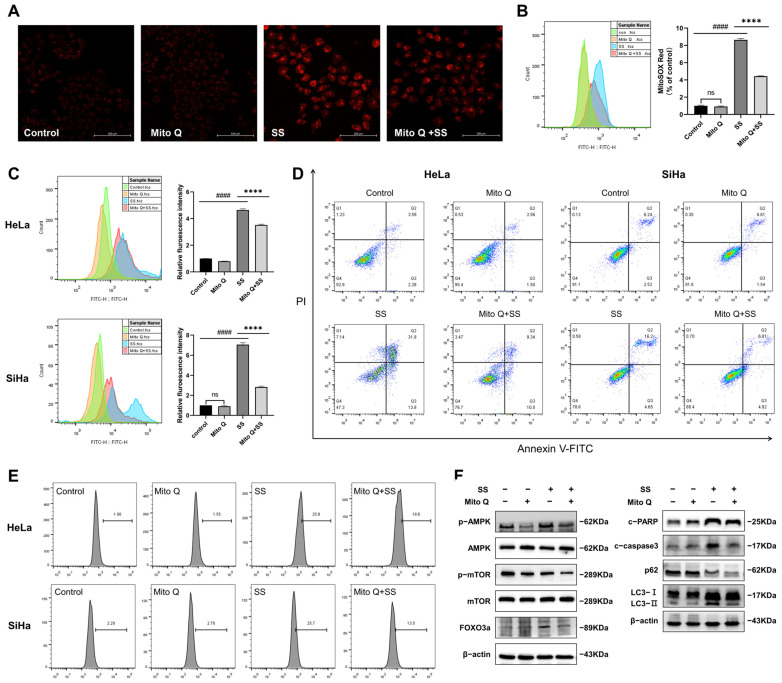
Sodium selenite induces autophagy and apoptosis via mitochondrial ROS. (**A**,**B**) After Mito Q pretreatment, mtROS levels were detected by confocal microscopy (**A**, magnification ×200) and quantified by flow cytometry (**B**) in HeLa cells. Changes in cell proliferation (**C**), the percentage of apoptotic cells (**D**), and the percentage of MDC-positive cells (**E**) were measured in HeLa and SiHa cells. (**F**) Expression levels of AMPK/mTOR/FOXO3a pathway proteins, as well as autophagy and apoptosis proteins in HeLa cells. All results were repeated at least three times. # indicates statistical significance compared to the control group, ns: *p* > 0.05, #### *p* < 0.0001; * indicates statistical significance compared to the SS treatment group, **** *p* < 0.0001. Con: Control; SS: sodium selenite; Mito Q: Mitoquinone mesylate; c-PARP: cleaved-PARP; c-caspase3: cleaved-caspase3.

To further elucidate the role of mtROS in SS-induced autophagy and apoptosis, we pretreated HeLa and SiHa cells with the mtROS inhibitor Mito Q. The results showed that Mito Q effectively reversed mtROS production in HeLa cells (Figure 6A,B). Furthermore, Mito Q alleviated SS-induced proliferation inhibition (Figure 6C) and the percentage of apoptotic cells (Figure 6D and Figure S4B) and significantly reduced the percentage of MDC-positive cells (Figure 6E and Figure S4C). Ad-mCherry-GFP-LC3B adenovirus transfection results also found that Mito Q pretreatment partially reversed green fluorescence quenching (Figure S4D). The expressions of LC3-II, cleaved-caspase3, cleaved-PARP, p-AMPK, and FOXO3a were partially reversed, but the inhibition of p-mTOR and p62 was not alleviated in HeLa cells (Figure 6F and Figure S4E). According to these findings, SS-induced autophagy and apoptosis within HeLa and SiHa cells may be induced by mtROS.

### 3.6. Inhibition of Autophagy Enhances Apoptosis in Cervical Cancer Cells

**Figure 7:**
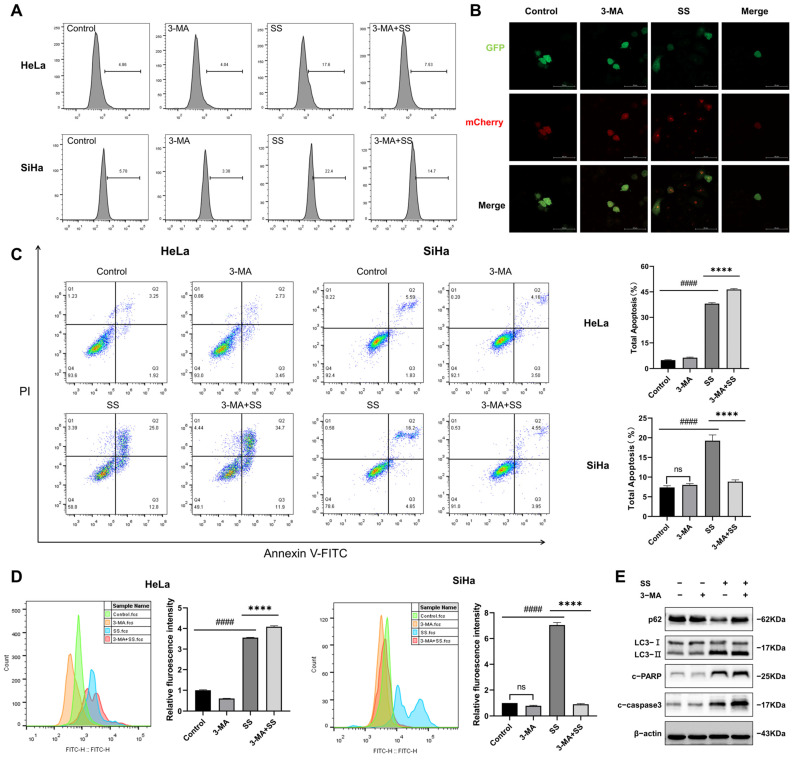
The effects of 3-MA pretreatment on sodium selenite-induced apoptosis and proliferation inhibition in HeLa and SiHa cells. After 3-MA pretreatment, changes in the percentage of MDC-positive cells (**A**), the percentage of apoptotic cells (**C**), and cell proliferation (**D**) in HeLa and SiHa cells. (**B**) Changes in autophagic flux in HeLa cells were detected using Ad-mCherry-GFP-LC3B, magnification ×400. (**E**) The expression of autophagy and apoptosis-related proteins was assessed by Western blot in HeLa cells. All results were repeated at least three times. # indicates statistical significance compared to the control group, ns: *p* > 0.05, #### *p* < 0.0001; * indicates statistical significance compared to the SS treatment group, **** *p* < 0.0001. 3-MA: 3-methyladenine; SS: sodium selenite; c-PARP: cleaved-PARP; c-caspase3: cleaved-caspase3.

Increasing evidence suggests that autophagy plays a significant role in both cellular protection and cytotoxicity. To investigate the relationship between SS-induced autophagy and apoptosis in HeLa and SiHa cells, we pretreated the cells with the autophagy inhibitor 3-MA. The results showed that, compared to the SS group, 3-MA significantly reduced the percentage of MDC-positive cells (Figure 7A and Figure S5A) and inhibited autophagic flux (Figure 7B). Moreover, in HeLa cells, we found that 3-MA further exacerbated SS-induced proliferation inhibition (Figure 7D) and promoted the percentage of apoptotic cells (Figure 7C). Meanwhile, compared to the SS group, the combined treatment of 3-MA and SS mitigated the upregulation of LC3-II and the downregulation of p62 but further increased the expression of cleaved-caspase3 and cleaved-PARP (Figure 7E and Figure S5B). However, unexpectedly, in SiHa cells, 3-MA pretreatment did not exhibit the same effects as in HeLa cells. Instead, it significantly inhibited SS-induced apoptosis (Figure 7C) and proliferation inhibition (Figure 7D).

### 3.7. SS Induces Autophagy and Apoptosis in Xenograft Tumors via the AMPK/mTOR/FOXO3a Signaling Pathway

**Figure 8:**
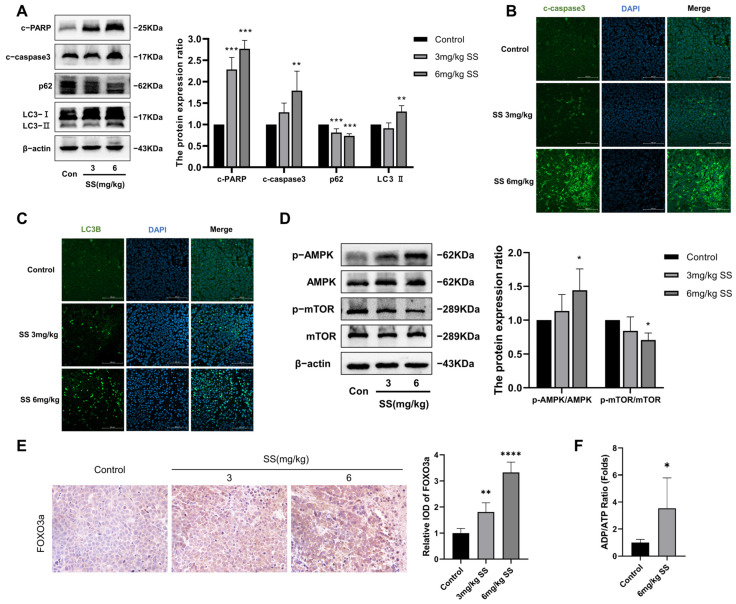
Sodium selenite induces autophagy and apoptosis in vivo via the AMPK/mTOR/FOXO3a signaling pathway. (**A**) Protein expression levels of LC3-II, p62, cleaved-caspase3, and cleaved-PARP in tumors from different intervention groups; the expression of β-actin was used as a reference. (**B**,**C**) Immunofluorescence analysis of cleaved-caspase3 and LC3B in tumor sections, magnification ×200. (**D**) Expression levels and quantitative analysis of p-AMPK, p-mTOR, and FOXO3a in tumors from different intervention groups; the expression of β-actin was used as a reference. (**E**) Immunohistochemical analysis of FOXO3a in tumor sections, magnification ×400. (**F**) Evaluation of changes in the ADP/ATP ratio in tumors. All results were repeated at least five times. * indicates statistical significance compared to the control group, * *p* < 0.05, ** *p* < 0.01, *** *p* < 0.001, **** *p* < 0.0001. Con: Control; SS: sodium selenite; c-PARP: cleaved-PARP; c-caspase3: cleaved-caspase3.

To investigate whether SS induces autophagy and apoptosis in HeLa xenograft tumors, we examined the expression levels of LC3-II, p62, cleaved-caspase3, and cleaved-PARP. Compared to the control group, SS significantly increased the expression of LC3-II, cleaved-caspase3, and cleaved-PARP while inhibiting the expression of p62 (Figure 8A). Immunofluorescence analysis of LC3B and cleaved-caspase3 confirmed these findings (Figure 8B,C). In vitro experiments suggested that the AMPK/mTOR/FOXO3a pathway may be a key regulatory pathway through which SS exerts its anticancer effects against CC. Therefore, we validated the expression of proteins related to the transplanted tumor using Western blot and immunohistochemistry. As shown in Figure 8D,E, SS intervention significantly increased the expression levels of p-AMPK and FOXO3a while decreasing the expression levels of p-mTOR. Additionally, SS intervention significantly increased the ADP/ATP ratio (Figure 8F).

## 4. Discussion

As a critical adaptive regulatory mechanism, autophagy helps cells and organisms cope with various endogenous or exogenous stresses [27,28]. Previous studies have shown that SS can induce autophagy in various cells [29,30]. However, there have been no reports on whether SS can induce autophagy in HeLa and SiHa cells. In this study, SS increased the expression of autophagy-related proteins both in vitro and in vivo, including an increased LC3-II protein ratio and p62 degradation, as well as promoting the percentage of MDC-positive cells in HeLa and SiHa cells. Consistent with our findings, Qian Jiang and Shin-Hyung Park also reported that SS induces autophagy in human leukemia and lung cancer cells [31,32]. Targeting apoptosis is considered a critical approach in anticancer drug development [33]. As a potential apoptosis modulator, SS has been reported to induce apoptosis in various cancer cells [34,35]. Our study demonstrated that SS intervention promoted apoptosis in HeLa and SiHa cells and HeLa xenograft tumors, accompanied by the upregulation of cleaved-caspase3 and cleaved-PARP protein expression. Additionally, there was no significant toxicity observed in the body weight, liver, or kidneys of the nude mice. These results suggested that the anti-tumor potential of SS may be achieved through the activation of autophagy and the induction of apoptosis.

AMPK acts as an energy sensor when cellular energy levels decline, primarily functioning in the mitochondria [25]. When cells undergo mitochondrial damage due to calcium overload, glucose starvation, or oxidative stress, AMPK is activated to restore energy homeostasis by promoting ATP-generating catabolic pathways [36]. The AMPK/mTOR signaling pathway has been identified as an upstream regulator of autophagy, where AMPK not only promotes the autophagic clearance of damaged lysosomes but also enhances the biogenesis of new lysosomal materials [23]. FOXO transcription factors regulate various biological processes, including cell cycle arrest, apoptosis, autophagy, and prevention of the accumulation of genetic toxicants and oxidative stress-induced damage [24,37]. Our results indicate that in HeLa and SiHa cells, SS disrupts intracellular energy balance by inhibiting glucose uptake, leading to AMPK phosphorylation, downregulation of p-mTOR/mTOR, and enhanced nuclear translocation and expression of FOXO3a. Pretreatment with compound C (AMPK scavenger) partially alleviated these changes and inhibited autophagy and apoptosis in HeLa and SiHa cells. These results were consistent with the findings of Hailing Yu et al., who also observed that SS induced autophagy and apoptosis through the AMPK/FOXO3a pathway in colorectal cancer cells [22]. Therefore, the anti-cervical cancer effect of SS may be mediated through the disruption of intracellular energy balance, which activates the AMPK/mTOR/FOXO3a pathway, thereby inhibiting cell proliferation and promoting apoptosis and autophagy.

Furthermore, SS treatment increased the production of mtROS. ROS serves as an important signaling molecule involved in information transmission through various cellular signaling pathways [38]. A significant body of research indicates that excessive ROS production induces autophagy and apoptosis in cancer cells by activating AMPK or other signaling pathways, thereby inhibiting tumor initiation and progression [39,40,41,42]. However, the specific role of mtROS in SS-induced autophagy and apoptosis in HeLa and SiHa cells remains unclear. Afterwards, this study examined whether mtROS production was an upstream event in the SS-mediated autophagy and apoptosis. Pre-treatment with Mito Q (a mtROS scavenger) significantly alleviated SS-induced mtROS production, cell apoptosis, cell proliferation, and autophagy levels in HeLa and SiHa cells. Besides, Mito Q pretreatment also abolished the effects of SS on the AMPK/mTOR/FOXO3a signaling pathway. Overall, findings from this work indicated that mtROS plays an important role in SS-mediated cell apoptosis, cell proliferation, and autophagy.

Apoptosis and autophagy are both critical for cell death [43]. The relationship between autophagy and apoptosis is generally categorized into three types: interdependence, conversion, and antagonism [44]. Under different conditions, autophagy can act as an antagonist to resist apoptosis or promote apoptosis [45,46]. To investigate the relationship between SS-induced autophagy and apoptosis in HeLa and SiHa cells, we pretreated HeLa and SiHa cells with 3-MA (an autophagy inhibitor). Surprisingly, the two cell lines exhibited completely opposite behaviors. In the HeLa cell line, 3-MA pretreatment further promoted SS-induced apoptosis and inhibition of cell proliferation. However, in the SiHa cell line, 3-MA completely reversed these effects. This suggests that in HeLa cells, autophagy may act as a stress response mechanism to help cells resist SS-induced apoptosis. In SiHa cells, SS-induced autophagy may directly or indirectly promote the apoptotic process, and therefore inhibiting autophagy with 3-MA reduced apoptosis.

In summary, this study elucidated the mechanisms by which SS inhibits CC progression. Our findings indicated that SS inhibits the proliferation of HeLa and SiHa cells by inducing autophagy and apoptosis. Further observations revealed that SS induces endoplasmic reticulum stress, leading to mitochondrial Ca^2+^ overload and mtROS accumulation while inhibiting glucose uptake, thereby impairing mitochondrial function. These effects further activated the AMPK/mTOR/FOXO3a pathway, ultimately resulting in apoptosis and autophagy. Additionally, we found that the activation of autophagy can protect HeLa cells to some extent from SS-induced apoptosis and inhibition of proliferation. However, in SiHa cells, autophagy is likely the main factor contributing to SS-induced apoptosis and inhibition of proliferation.

Although our study revealed the significant role of SS in inhibiting CC, our study observed that pretreatment with 3-MA resulted in completely opposite effects of SS-induced apoptosis and proliferation inhibition in HeLa and SiHa cells. The underlying mechanisms for these opposing effects were not investigated in this study. Future research will aim to explore the potential mechanisms behind these observations.

## Data Availability

Data will be made available upon request from the corresponding authors.

## Supplementary Materials

The following supporting information can be downloaded at: https://www.mdpi.com/article/10.3390/antiox13081004/s1. Figure S1: GO terms in biological processes, molecular functions, and cellular components showing significant differences; Figure S2: Sodium selenite induces autophagy and apoptosis in HeLa and SiHa cells; Figure S3: The AMPK/mTOR/FOXO3a signaling pathway is the key pathway involved in sodium selenite -induced autophagy and apoptosis; Figure S4: Sodium selenite induces autophagy and apoptosis via mitochondrial ROS; Figure S5: The effects of 3-MA pretreatment on sodium selenite-induced apoptosis in HeLa cells.
