# Supplementary material for: Sodium Selenite Induces Autophagy and Apoptosis in Cervical Cancer Cells via Mitochondrial ROS-Activated AMPK/mTOR/FOXO3a Pathway
Source: Antioxidants (Basel). 2024 Aug 19;13(8):1004. doi: 10.3390/antiox13081004 (PMC11352036; doi:10.3390/antiox13081004)
Supplement: Supplementary file 1 [file antioxidants-13-01004-s001.zip › antioxidants-3089590-supplementary.pdf]

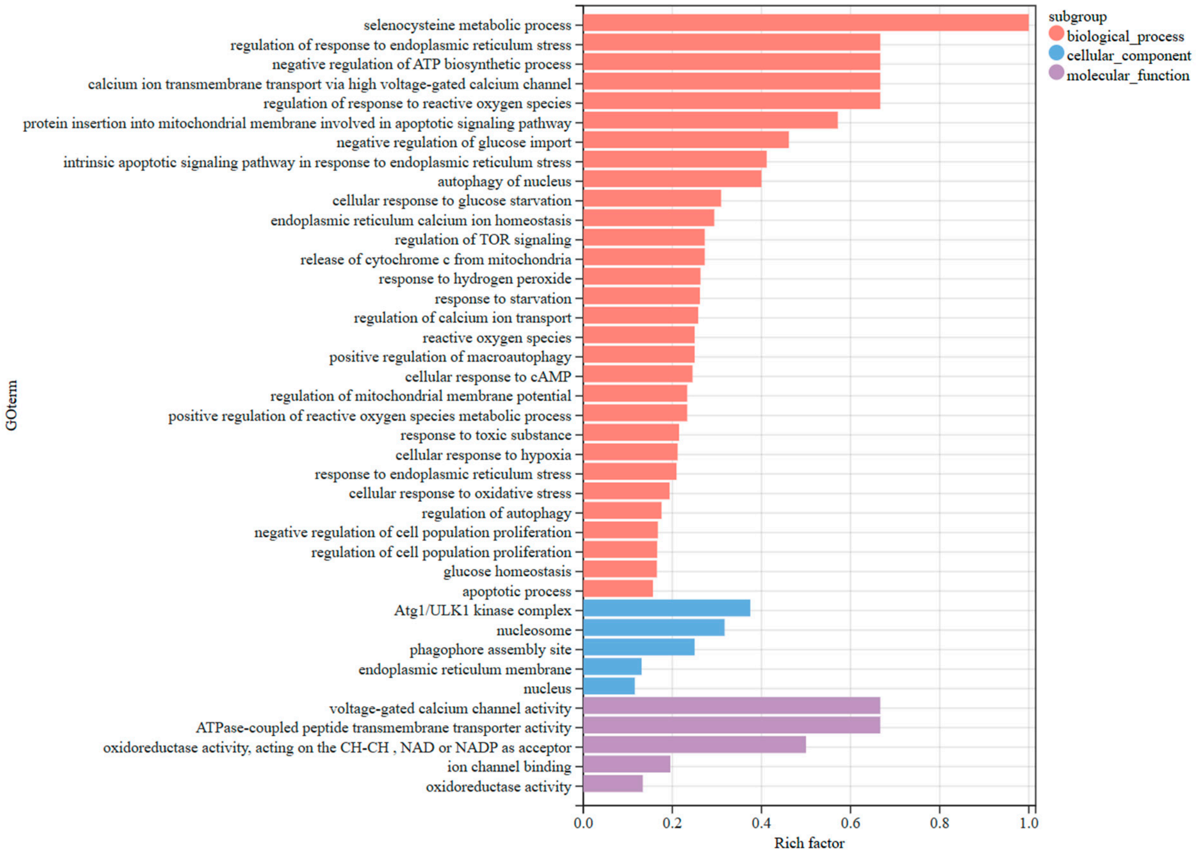

Figure S1. GO terms in biological processes, molecular functions, and cellular components showing significant differences.

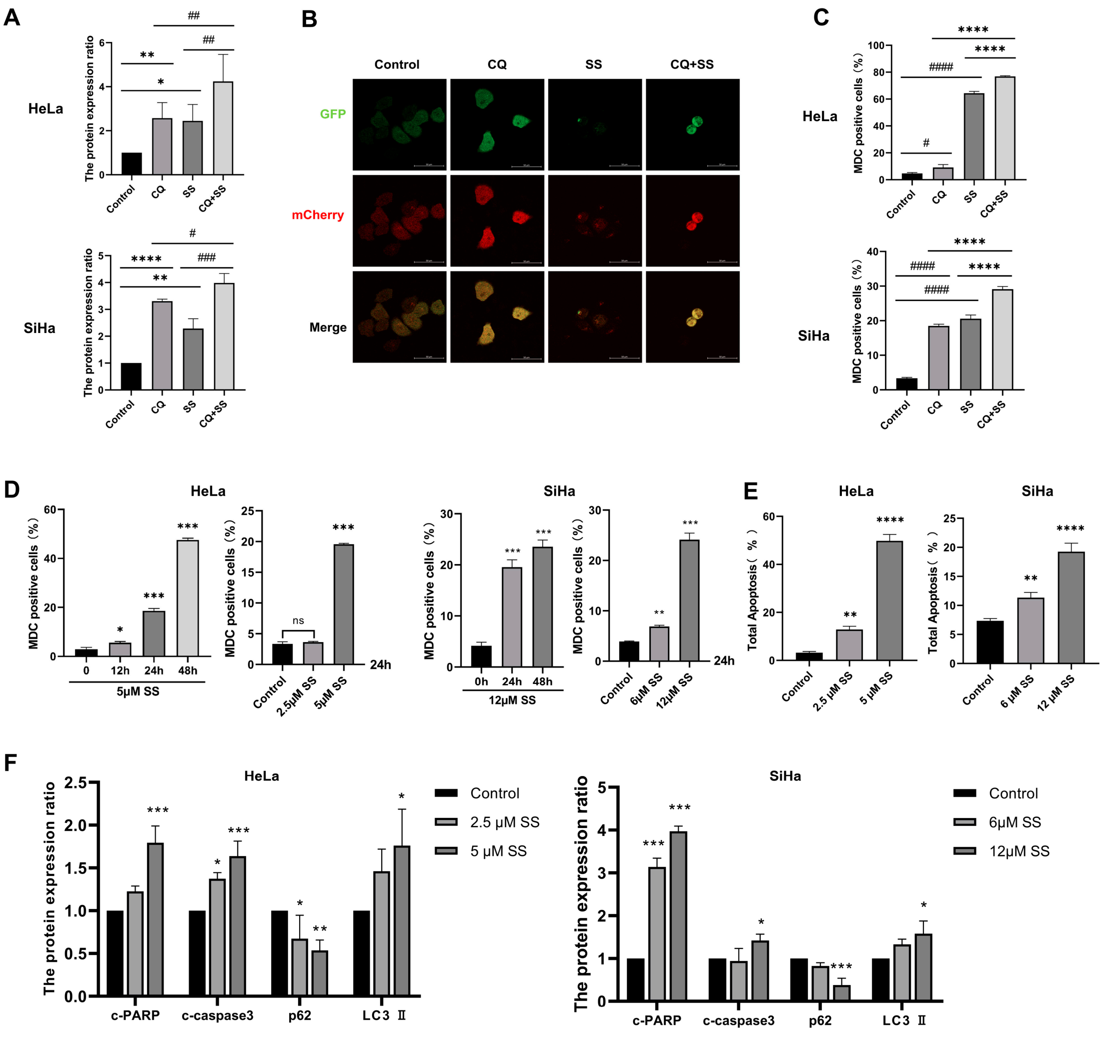

Figure S2. Sodium selenite induces autophagy and apoptosis in HeLa and SiHa cells. (**A**) The expression of LC3−II in HeLa and SiHa cells after CQ pretreatment (**B**) Observation of autophagosome and lysosome fusion in Ad−mCherry−GFP−LC3B−transfected HeLa cells by monitoring GFP fluorescence quenching after CQ pretreatment, magnification ×400. (**C**) Bar chart of the quantitative analysis of the change in the percentage of MDC−positive cells after CQ pretreatment (**D**) Bar chart of the quantitative analysis of the percentage of MDC−positive cells after SS treatment at different times and doses in HeLa and SiHa cells. (**E**) Bar chart of the quantitative analysis of the percentage of apoptotic cells after different doses SS treatment in HeLa and SiHa cells. (**F**) Bar chart of the quantitative analysis of autophagy (LC3−II and p62) and apoptosis (cleaved−caspase3 and cleaved−PARP) protein expression in HeLa and SiHa cells after treatment with different doses of SS, the expression of β−actin was used as a reference. All experiments were repeated at least three times. * indicates statistical significance compared to the control group, **p* < 0.05, ***p* < 0.01, ****p* < 0.001, *****p* < 0.0001; # indicates statistical significance compared to the CQ+SS co−treatment group, #*p* < 0.05, ##*p* < 0.01, ###*p* < 0.001, ####*p* < 0.0001. CQ: Chloroquine diphosphate salt; SS: sodium selenite; c−PARP: cleaved−PARP; c−caspase3: cleaved−caspase3.

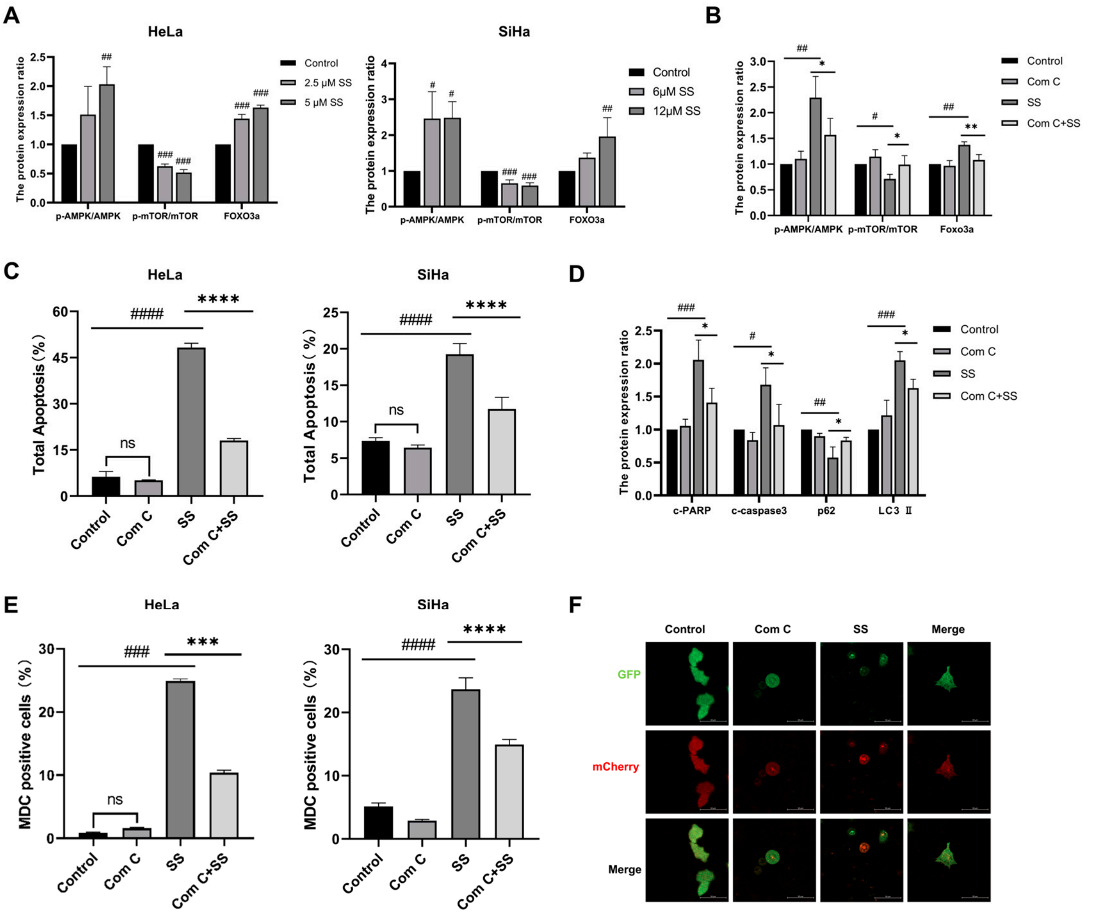

Figure S3. The AMPK/mTOR/FOXO3a signaling pathway is the key pathway involved in sodium selenite -induced autophagy and apoptosis. (**A**) Bar chart of the quantitative analysis of AMPK/mTOR/FOXO3a pathway proteins expression in HeLa and SiHa cells after treatment with different doses of SS, the expression of β−actin or Tubulin was used as a reference. (**B**) Bar chart of the quantitative analysis of the expression of AMPK/mTOR/FOXO3a pathway proteins after compound C pretreatment in HeLa cells, the expression of β−actin was used as a reference. (**C**) Bar chart of the quantitative analysis of the percentage of apoptotic cells in HeLa and SiHa cells after compound C pretreatment. (**D**) Bar chart of the quantitative analysis of the expression of autophagy and apoptosis proteins after compound C pretreatment in HeLa cells, the expression of β−actin was used as a reference. (**E**) Bar chart of the quantitative analysis of the change in the percentage of MDC−positive cells after compound C pretreatment. (**F**) Monitoring changes in autophagic flux by confocal microscopy in HeLa cells, magnification ×400. All experiments were repeated at least three times. # indicates statistical significance compared to the control group, ns: $p > 0.05$, #$p < 0.05$, ##$p < 0.01$, ###$p < 0.001$, ####$p < 0.0001$; * indicates statistical significance compared to the SS treatment group, *$p < 0.05$, **$p < 0.01$, ***$p < 0.001$, ****$p < 0.0001$. Com C: Compound C; SS: sodium selenite; c−PARP: cleaved−PARP; c−caspase3: cleaved−caspase3.

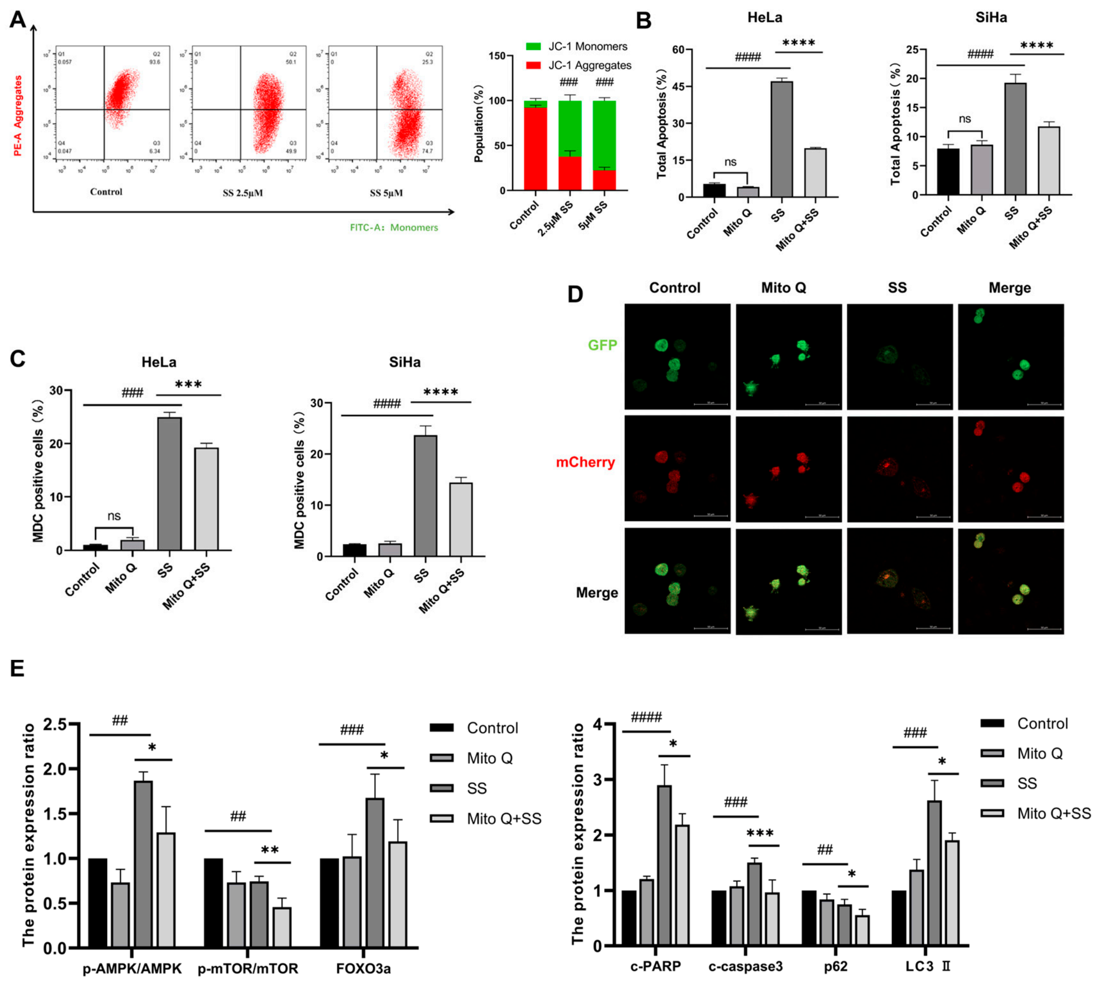

Figure S4. Sodium selenite induces autophagy and apoptosis via mitochondrial ROS. (**A**) Detection of the effects of different doses of SS on mitochondrial membrane potential changes in HeLa cells by flow cytometry. (**B**) Bar chart of the quantitative analysis of the percentage of apoptotic cells after Mito Q pretreatment in HeLa and SiHa cells. (**C**) Bar chart of the quantitative analysis of the percentage of MDC−positive cells after Mito Q pretreatment in HeLa and SiHa cells. (**D**) Monitoring changes in autophagic flux by confocal microscopy in HeLa cells, magnification ×400. (**E**) Bar chart of the quantitative analysis of AMPK/mTOR/FOXO3a pathway proteins, autophagy and apoptosis proteins expression after Mito Q pretreatment in HeLa cells, the expression of β−actin was used as a reference. All experiments were repeated at least three times. # indicates statistical significance compared to the control group, ns: p > 0.05, ##$p < 0.01$, ###$p < 0.001$, ####$p < 0.0001$; * indicates statistical significance compared to the SS treatment group, *$p < 0.05$, **$p < 0.01$, ***$p < 0.001$, ****$p < 0.0001$. Mito Q: Mitoquinone mesylate; SS: sodium selenite; c−PARP: cleaved−PARP; c−caspase3: cleaved−caspase3.

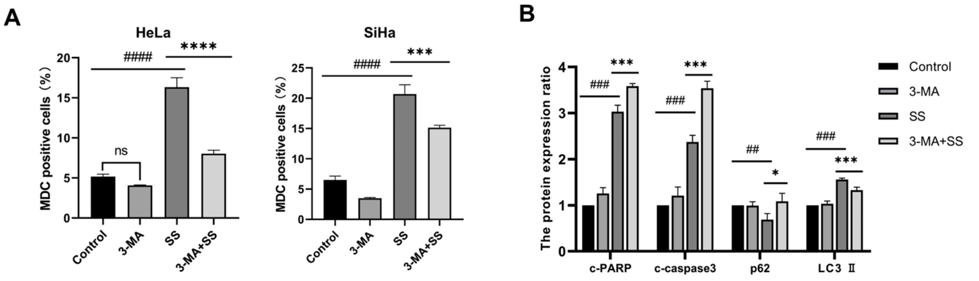

Figure S5. The effects of 3−MA pretreatment on sodium selenite−induced apoptosis in HeLa cells. (**A**) Bar chart of the quantitative analysis of the percentage of MDC−positive cells after 3−MA pretreatment in HeLa and SiHa cells. (**B**) Bar chart of the quantitative analysis of the expression of autophagy and apoptosis proteins after 3−MA pretreatment in HeLa cells, the expression of β−actin was used as a reference. All experiments were repeated at least three times. # indicates statistical significance compared to the control group, ns: $p > 0.05$, ##$p < 0.01$, ###$p < 0.001$, ####$p < 0.0001$; * indicates statistical significance compared to the SS treatment group, *$p < 0.05$, ***$p < 0.001$, ****$p < 0.0001$. 3−MA: 3−methyladenine; SS: sodium selenite; c−PARP: cleaved−PARP; c−caspase3: cleaved−caspase3.
